# HOPITAL DE VERSAILLES

**Published:** 1830-02

**Authors:** 


### HOPITAL DE VERSAILLES.

### *Aneurism of the left Common Carotid, cured by Ligature.*

A soldier, named Antoine Bolfing, ætat. forty, related that, a month before his admission, while he was playing with one of his comrades, he was roughly seized by the throat. He experienced great pain at the moment: and, since this occurrence, he perceived a swelling in the part, which continued to increase in size. M. Noble the head surgeon of the hospital directed the patient to be treated according to the plan of Valsalva. Leeches were freely applied, and the tumor was covered with ice, &c.

Notwithstanding the adoption of these means, the tumor daily increased, and at length produced so much distress, by pressing upon the larynx and œsophagus, that the patient urgently requested to be relieved by some more effectual treatment. The tumor was situated at the upper part of the left side of the neck: it extended from the mastoid process to the middle of the neck, opposite the base of the larynx, aud transversely from the angle of the jaw to the symphysis of the chin, throwing the sterno-cleido-mastoideus outwards, and pressing back the larynx. Throughout the whole extent of the tumor, a strong pulsation was perceptible, which was synchronous with the action of the artery at the wrist. The distended skin preserved a natural appearance. Pressure below the tumor caused the pulsation in it to cease, and sensibly diminished its volume. The general state of the patient was favorable: he complained only of pain on the left side of the head, and of great difficulty in swallowing and breathing. In other respects he was in good health, very anxious for an operation, and with a firm determination to support it with courage. On the 20th November it was performed in the following manner.

The patient was placed upon a bed, his head and trunk being elevated. M. Maurin made an incision about three inches long, in the direction of the anterior margin of the sterno-cleido-mastoideus, from the inferior part of the tumor down to the clavicle. The cellular tissue laying between the sterno-mastoid and the sterno-hyoideus muscles was then carefully divided. The omo-hyoideus was raised upwards with a spatula, with which was also divided the sheath formed by the cellular tissue which envelopes the external jugular vein, the pneumo-gastric nerve, and carotid artery. The carotid was completely insulated from the surrounding parts, and with a female catheter, armed with a double thread, *two* ligatures were placed under it. Upon tying the ligature, the pulsation of the tumor entirely ceased. One ligature was tied an inch below the part where the omo-hyoideus muscle passes over the artery, and the other upon a level with that muscle. Not more than an ounce of blood was lost during the operation, which was not retarded by any accidental occurrence. The wound was immediately closed, the ligatures being left hanging out.

One hour after the operation, the patient was calm; pulse sixty-five. In four hours the inferior extremities were cold, and there was a sensation of oppression and tightness in the epigastric region, with some pain in the left arm; pulse sixty-eight. No pulsation in the tumor, the size of which was much lessened.—An ethereal mixture. The inferior extremities to be rubbed, and covered with flannel.

At seven o'clock in the evening, eight hours after the operation, there were general sweat, cough, frequent expectoration of mucus, great heat of skin, pulse sixty-eight. At nine o'clock, great oppression in the epigastrium, intense headach, face flushed on the right side, pulse quick.—Twelve leeches to be applied to the epigastrium.

21st.—Has been quiet during the night, but had no sleep. Oppression diminished, expectorates more freely, pulse hard and frequent, face flushed. In the evening, his cough increased, pulse eighty; pain in the head and epigastrium increased.—To be bled to eight ounces, and to take a pectoral mixture, with syrup of poppies.

22d.—Has passed a sleepless night, is much oppressed; great pain in the epigastrium, headach. Pulse hard, and 100; throbbing of the left temporal artery. The unpleasant odour and abundance of the suppuration rendered it necessary to remove the dressings. The centre of the wound was superficially united.

Evening: V.S. from the arm. Emulsion continued.

Until the 30th, the patient went on well. The tumor had much diminished in size. The wound looked healthy, suppuration less abundant, and of good quality. The upper ligature came away on the 29th.

December 1st.—Has had a bad night; pain and oppression in the epigastrium returned. Cough frequent, and without expectoration. Pulse ninety-five, and hard.

2d.—Better in every respect. An enema was given, as the bowels had not been open.

From this time no remarkable symptoms occurred. On the 15th December, the wound was entirely healed; the tumor was reduced to about the size of a small hen's egg. At the time the case was reported, the patient had not left the hospital, as he was affected with slight catarrh, which he had caught by walking about the wards.—*Revue Medicale.*

The manner in which aneurism of the carotid was caused in the above case is somewhat singular, although the effect produced by the operation leaves no room to doubt the nature of the disease. To the application of *two* ligatures, we decidedly object: we thought, indeed, that this practice had been entirely banished from modern surgery, and it appears evident that in this instance it was injurious, by interrupting the favorable union of the wound. Experience has amply proved that *one* ligature is quite as effectual, and quite as safe, in every respect as two.—EDITOR.

